# Osteoid Osteoma of the Proximal Femur: Pitfalls in Diagnosis and Performance of Open Surgical Resection

**DOI:** 10.3389/fsurg.2022.922317

**Published:** 2022-06-28

**Authors:** Hao Zeng, Hongbo He, Xiaopeng Tong, Zhiwei Wang, Rongsheng Luo, Qing Liu

**Affiliations:** ^1^ Department of Orthopaedics, Xiangya Hospital, Central South University, Changsha, Hunan, China; ^2^ National Clinical Research Center for Geriatric Disorders, Xiangya Hospital, Changsha, Hunan, China

**Keywords:** osteoid osteoma, proximal femoral, misdiagnosis, thin-layer CT, open surgery

## Abstract

**Aims:**

Proximal femoral osteoid osteoma (OO) is extremely easy to be misdiagnosed or missed. The purpose of this study was to retrospectively analyze the clinical data of patients with proximal femoral OO in order to determine the clinical manifestation and imaging characteristics of the disease, so as to provide help for the preoperative diagnosis and clinical treatment of proximal femoral OO.

**Methods:**

This was a retrospective study involving 35 patients with proximal femoral OO admitted into our hospital from January 2015 to January 2021. The baseline characteristics of the participants included; 24 males and 11 females, aged between 13 and 25 (mean 16.2) years old, and the course of the disease was 1 to 14 (mean 6.3) months. We used previous medical experience records of the patients to analyze for the causes of misdiagnosis. Moreover, we compared the difference between preoperative and postoperative treatment practices in alleviating pain in OO patients and restoring hip function. Follow-ups were carried out regularly, and patients advised to avoid strenuous exercises for 3 months.

**Results:**

We followed up 35 patients (25 intercortical, 4 sub-periosteal, and 6 medullary) for an average of 41.4 months. We found that 15 patients (42.9%) had been misdiagnosed of synovitis, perthes disease, osteomyelitis, intra-articular infection, joint tuberculosis and hip impingement syndrome, whose average time from symptoms to diagnosis were 6.3 months. Postoperative pain score and joint function score improved significantly compared with preoperative, and complications were rare.

**Conclusion:**

Open surgical resection constitutes an effective treatment for proximal femoral OO by accurately and completely removing the nidus. Wrong choice of examination, and the complexity and diversity of clinical manifestations constitutes the main reasons for the misdiagnosis of proximal femoral OO.

## Introduction

**Figure 1:**
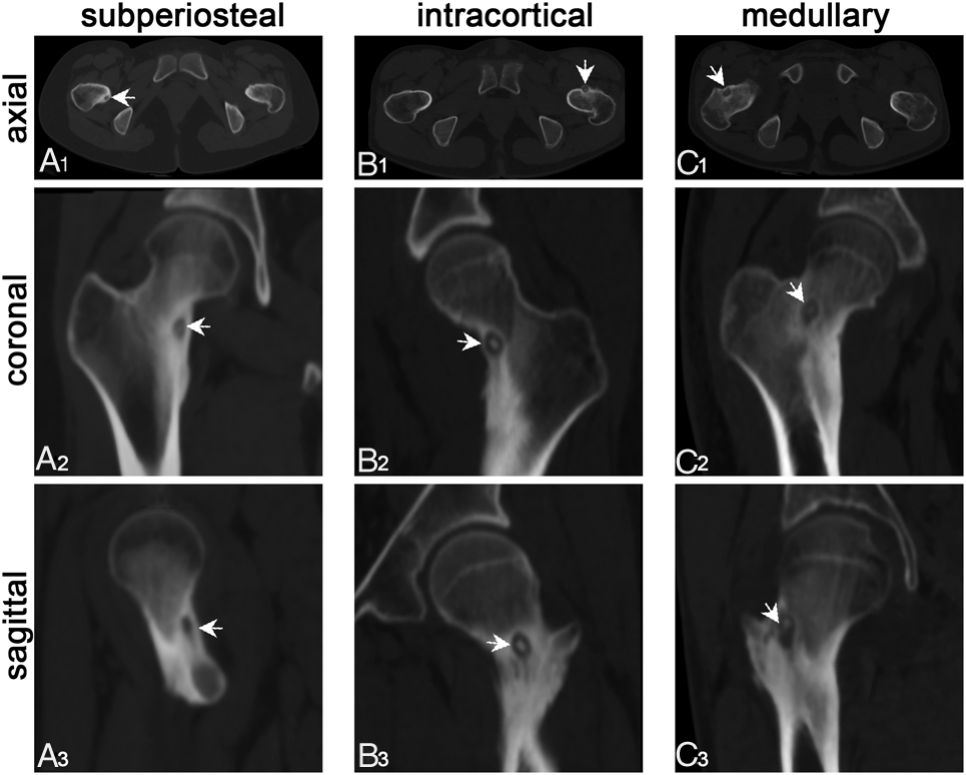
Oo was classified into sub-periosteal, intra-cortical, and medullary type based on the location of the nidus using thin-layer CT. (**A1–A3**) Sub-periosteal: the nidus is located under the periosteum and outside the cortex; (**B1–B3**) intra-cortical: the nidus is located inside the bone cortex and expands inwards and outwards; (**C1–C3**) medullary: the nidus is completely intramedullary. The white arrow shows the nidus, some of which have high-density calcification.

Osteoid osteoma (OO) constitutes a benign osteogenic tumour typified by persistent blunt pain with nocturnal aggravation that is relieved by oral nonsteroidal anti-inflammatory drugs (1, 2). OO is characterized by a round nidus of tumour tissue with a diameter of less than 2 cm, mostly less than 1 cm, and the lesion is composed of bone-like tissue, rich in blood vessels, and cells (3). The morbidity of OO accounts for about 10% of benign bone tumors, which accounts for 2% to 3% of all primary bone tumors. OO most frequently affects individuals in their second and third decades of life with significantly higher morbidity in males compared to females, and the ratio ranges between 2:1 and 3:1 (4, 5). It predominantly occurs in the cortex of the long tubular bone, particularly in the femur and tibia, with the proximal femoral more frequently involved. OO is classified into sub-periosteal, intra-cortical, and medullary based on the position of the nidus within the bone (Figure 1). Intra-cortical OO is the most common, representing approximately 75% of the lesions. On the contrary, medullary OO is relatively rare and typically juxta-articular in location, it's less reactive bone around the nidus complicates diagnosis (6, 7).

The hip joint is the joint with the greatest mobility of the human lower limbs, and the proximal femur is the prone site of bone and soft tissue tumors. Because it is adjacent to the hip joint, the diseases in this site often show inconsistent clinical manifestations (6, 7). Therefore, OO occurring at the hip joint presents with atypical clinical symptoms and imaging manifestations, so it is easy to be misdiagnosed or missed, and patients often suffer unnecessary treatment due to delayed diagnosis, resulting in great physical suffering and financial burden (5, 8, 9).

Due to the absence of periosteum in the hip capsule, there is little or no reactive sclerotic bone formation in intraarticular OO on conventional radiographs, and the lesion can be detected in less than 50% of cases with a diameter of less than 3 mm (2, 10). The most common signs in patients with proximal femur are claudication, reduced range of motion of the hip, and positive signs of hip impingement (90 degree internal hip flexion and mild adduction pain) (5). Major differential diagnoses include synovitis of the hip, intra-articular infection, Perthes disease, Brodie's abscess, chondroblastoma, and acetabular impingement syndrome (11–14). The main treatment methods for OO are radiofrequency ablation (RFA) and surgical resection. In recent years, with the development of precision medicine and navigation technology, RFA has played a dominant role in the clinical treatment of OO. It is indeed an excellent choice for the treatment of OO with less trauma, quick recovery and excellent efficacy (15, 16). However, there are still some controversies over the treatment of proximal femoral OO considering the particular location of the nidus (17, 18). The nidus of the proximal femur is often adjacent to the femoral sheath, and there are the main feeding vessels of the femoral head on the inside of the articular capsule. The hyperthermia caused by RFA is easy to damage blood vessels and nerves, soft tissue necrosis and bone necrosis around the diameter of 1 cm, so there may be secondary serious inflammatory reaction, pathological fracture and long-term avascular necrosis of the femoral head (19, 20). Open surgical curettage can accurately curettage tumor nidus and obtain pathological results. At the same time, it can be given selectively according to the size of lesions to prevent the occurrence of postoperative bone related complications (21). Up to now, there is still no unified standard for the clinical treatment of proximal femoral OO.

In the current study, we retrospectively analyzed the clinical data of patients with OO of the proximal femur treated in our tumor center in recent 6 years, and evaluated the clinical efficacy of open surgery from the perspective of diagnosis, treatment and complication follow-up. The purpose is to share the clinical experience of our tumor center in the diagnosis and treatment of proximal femur OO, and provide a research basis for the standardized treatment of the disease, in order to achieve rapid and accurate diagnosis and optimize the treatment strategy of the proximal femur of OO.

## Materials and Methods

### Patients

According to the approval of the ethics committee of Xiangya Hospital, follow-up analysis was performed on the patients with OO treated in our hospital, and the data of 114 consecutive patients admitted in our hospital from January 2015 to January 2021 were analyzed. The inclusion criteria constituted; pathological confirmation of OO, location of the nidus in the proximal femoral, open surgical resection was used in the treatment, complete follow-up data with a follow-up time of more than 12 months. The exclusion criteria composed; OO patients receiving other surgical treatments other than open surgical resection, less than 12 months of follow-up or incomplete follow-up data. All patients participating in the study received informed consent and signed consent from the patient or their legal guardians.

### Preoperative Diagnosis and Evaluation

**Figure 2:**
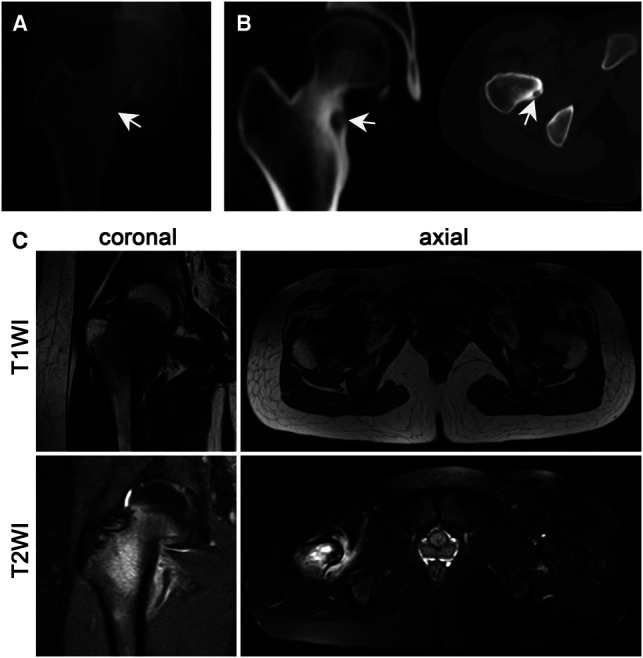
Imaging examination significantly helped in the diagnosis of OO. (**A**) The radiography examination shows a round and transparent area. (**B**) Thin-layer CT accurately displays the nidus and the surrounding reactive hyperplastic bone tissue (the white arrow shows the nidus). (**C**) MRI examination lacks specificity, T1WI shows low to moderate signal intensity and T2WI shows moderate to high signal intensity.

In this study, all patients underwent preoperative X-ray and thin-slice CT scans, 28 patients underwent MRI and 8 patients underwent radionuclide bone scanning. Pain is almost the only symptom in the early stage of OO. Most of the pain is persistent and nocturnal, which can be relieved by non steroidal anti-inflammatory drugs (NSAIDs). The patients in this group had a clear history of nocturnal pain and self-reported NSAIDs drug treatment was effective. Typical radiography characteristics of OO include fusiform sclerosis of the cortex centered on the nidus, which is a transparent area with a diameter of less than 2 cm (Figure 2A). Computed tomography (CT) can accurately locate the intraosseous lesions and periosteal reaction areas (Figure 2B). Due to the lack of characteristic signal and the relatively low spatial resolution of magnetic resonance imaging (MRI) (22), which is mostly used to determine the perilesional bone marrow edema (23), medial retinacular thickening, diffuse synovitis in the hip joint, joint effusion, etc (Figure 2C). According to the results of preoperative thin-layer CT scanning, three types of OO were defined according to the location of low-density tumor nidus: ① Sub-periosteal: the nidus is located under the periosteum and outside the cortex; ② intra-cortical: the nidus is located inside the bone cortex and expands inwards and outwards; ③ medullary: the nidus is completely intramedullary. Meanwhile, with the attachment point of proximal femoral hip joint capsule as the boundary, the nidus above the attachment point are defined as intra-capsular type, and the rest are extra-capsular type.

Demographic and clinical information was recorded before all procedures, including symptoms, time from symptoms to diagnosis, physical examination, misdiagnosis, and imaging findings. NSAIDs treatment was given to all patients before operation, and a visual analogue scale (VAS) (24) was used to evaluate pain pre and post-treatment. We used the modified Harris score system to evaluate hip joint function (10).

### Procedure

**Figure 3:**
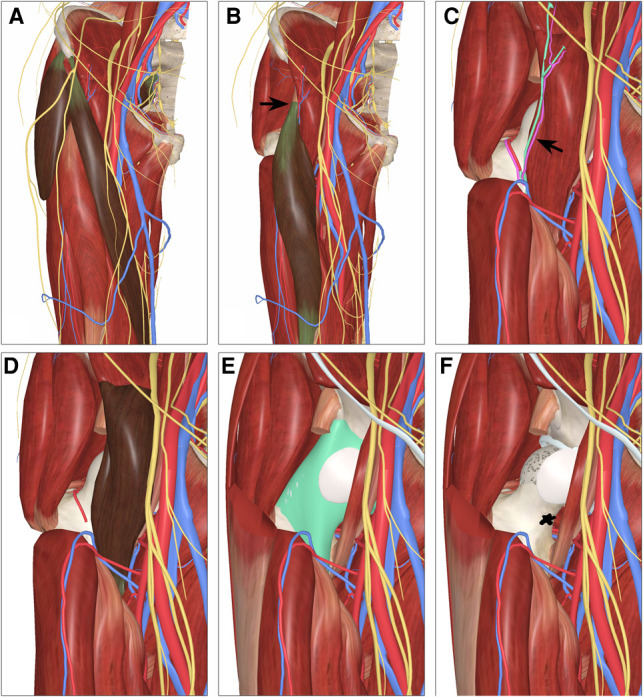
Procedure of simth-petersen (SP) approach for open surgery. (**A**) Expose along the gap between tensor fascia lata and sartorius muscle; (**B**) Expose the rectus femoris; (**C**) Ligate the ascending branch of the lateral femoral circumflex artery; (**D**) Pull the iliopsoas inward; (**E**, **F**) Cut the joint capsule and rotate the lower leg to expose the focus.

Considering that the proximal femoral OO is mostly located in the femoral neck or lesser trochanter, and some cases are located in the joint capsule, the Simth-Petersen (SP) approach was chosen for lesion resection. Enter along the gap between the tensor fascia lata and sartorius (Figure 3A), pay attention to protect the lateral femoral cutaneous nerve, expose the rectus femoris muscle (Figure 3B), then pull the rectus femoris muscle laterally, ligate the ascending branch of the external circumflex artery (Figure 3C), and then pull the rectus femoris muscle laterally, the iliopsoas muscle is pulled medially to expose the joint capsule (Figures 3D,E). A C-arm machine was used to locate the lesion, and the lower extremity was adducted and externally rotated to reveal the lesion (Figure 3F). For intra-articular lesions, we use a T-shaped incision of the joint capsule. After accurately locating the lesion, high-speed burr was used to remove the hyperplastic reaction bone on the surface, and the pathological tissue was scraped with a curette for pathological examination. Subsequently, the boundary of the curettage was expanded with a high-speed burr, and after thoroughly irrigating the surgical field, the periphery of the lesion was cauterized with an electric knife to ensure complete removal of the lesion. Bone grafting was performed for lesions with large bone defects, and preventive internal fixation was performed for some patients with large defects in the femoral calcar. Finally, the incision was sutured successively and a drainage tube was placed. We intravenously administered antibiotics until the drainage tube was removed to encourage early postoperative activity. The patients used crutches for walking within 1 month and avoided strenuous exercise within 3 months after the operation.

### Follow-up and Evaluation

Patients were followed-up radiographically every 3 months for the first 2 years, every 6 months until the 5th year, and annually after that. The follow-up involved the evaluation of postoperative pain, joint function, and recording the occurrence of complications.

### Statistical Analysis

SPSS 21.0 (SPSS Inc., IBM, Chicago) statistical software was used in data analysis. The quantitative data were expressed by mean ± standard deviation. Preoperative and postoperative VAS pain scores and modified Harris scores were analyzed using the paired *T*-test. *P* value ≤ 0.05 indicated statistical significance.

## Results

### Demographic and Clinical Record

We enrolled 35 patients into this study, with an average age of 16.2 ± 3.4 years (13–25 years), comprising of 24 males and 11 females (Supplementary table). The radiography results of 29 cases revealed an increase in local density and different degrees of cortical thickening, and 6 cases had low-density nidus. Thin-slice CT scans revealed nidus across all the patients, in which twenty-five were intra-cortical, 6 medullary, and 4 sub-periosteal. 15 patients with calcification in the lesion had the bull's eye sign changes. 31% of the lesions were intracapsular and 69% were extracapsular. The first symptoms reported by patients included hip pain with evident nocturnal pain. The VAS scores were 5.6 ± 1.0 in preoperative un-administered NSAIDs status. The average duration in this group was 6.3 ± 3.6 months (1–14 months), 5 patients complained of ipsilateral knee joint, 9 patients had lameness preoperative, 3 patients had atrophy of limb muscles, and the average Harris score of hip joint preoperative was 55.6 ± 10.9 points (Table 1).

**Table 1:** Demographic and clinical information of patients.

Gender			
M		24 (68.6%)	
F		11 (31.4%)	
Age(years)		16.2 ± 3.4	
Classification			
subperiosteal		4 (11.4%)	
intracortical		25 (71.4%)	
medullary		6 (17.1%)	
Localization			
intra-articular		11 (31.4%)	
extra-articular		24 (68.6%)	
Duration of symptom (months)		6.3 ± 3.7	
Misdiagnosis		15	
synovitis		3 (6.9%)	
perthes disease		1 (2.9%)	
osteomyelitis		3 (8.6%)	
intra-articular infection		2 (5.7%)	
joint tuberculosis		1 (2.9%)	
hip impingement syndrome		5 (14.3%)	
Follow-up time(months)		41.4 ± 14.6	
Comparative analysis	Preoperative	Postoperative	*P* value
VAS score	5.6 ± 1.0	0.5 ± 0.7	*P* < 0.001
Harris score	55.6 ± 10.9	99.5 ± 1.0	*P* < 0.001

### Misdiagnosis

In this group, 15 cases were initially diagnosed in other hospitals, of which 6 patients visited more than three hospitals. These patients were misdiagnosed at the first diagnosis. Misdiagnosis of synovitis and sclerosing osteomyelitis were reported in three cases each, Perthes disease and joint tuberculosis in one case each, intra-articular infection in two cases, hip impingement syndrome in five cases. All patients had a history of nocturnal pain, and the pain could be relieved by taking NSAIDs, five patients had taken hormone and immunosuppressive drugs, one patient had received anti-tuberculosis treatment, nine patients had received surgical treatment, seven patients had acupuncture and physical therapy in traditional Chinese medicine (TCM).

### Treatment

**Figure 4:**
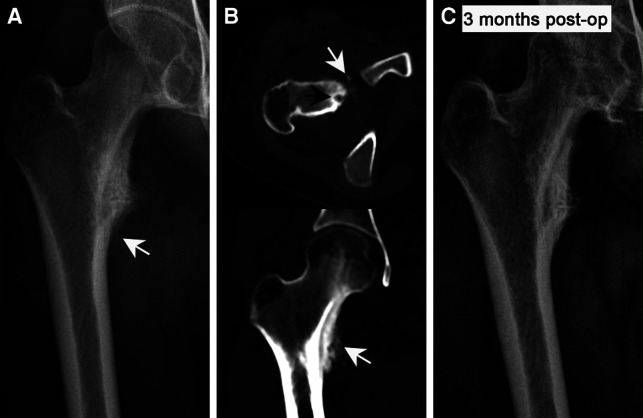
The preoperative and postoperative imaging characteristics of OO in a lesser trochanter. (**A**) The radiography shows the periosteal reaction caused by the nidus but the nidus is not visible. (**B**) Thin-layer CT accurately displays the nidus and the surrounding hyperplastic bone. (**C**) Postoperative radiographs show that the reaction bone was completely absorbed and the bone morphology was restored to normal (The white arrow shows the nidus, black arrows indicate reactive hyperplastic bone).

**Figure 5:**
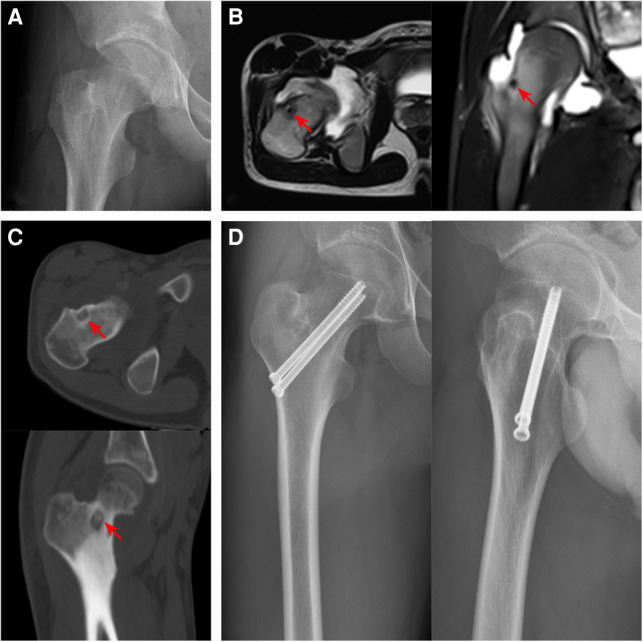
Preoperative and postoperative imaging features of femoral neck OO. (**A**) The radiography showed a light transmission low-density lesion in the center of the base of the femoral neck. (**B**) The T2WI image of MRI showed low signal intensity, and the edema signal around the lesion was not obvious. (**C**) Thin-slice CT showed an osteolytic lesion in the anterolateral medulla at the base of the femoral neck with scattered calcification. (**D**) The radiography findings after curettage and bone grafting combined with cannulated screw prophylactic internal fixation (The Red arrows indicate lesions).

Patients were treated with expanded curettage *via* SP approach. 29 patients could see clear tumor nests during the operation, scrape sediment like tumor tissue for pathological examination, 6 patients underwent local lesion resection (Figure 4), and 8 patients underwent preventive internal fixation (Figure 5). The original pain caused by tumor disappeared within 24 h after operation. The average postoperative VAS score was 0.5 ± 0.7.

### Follow up and Evaluation

The average follow-up time was 41.4 ± 14.6 months (24–81 months). At the first follow-up, the pain caused by the original tumor disappeared, and there was no report of pain recurrence. The average Harris score after hip surgery was 99.5 ± 1.0. During the perioperative period, 3 patients had delayed wound healing and 5 patients had symptoms of lateral femoral cutaneous nerve pain. After symptomatic treatment, the symptoms of the above patients were completely relieved, and there were no serious complications requiring reoperation. No complications such as hip infection, avascular necrosis of femoral head, femoral neck fracture and deep venous thrombosis occurred during follow-up. In addition, no superficial sensory abnormalities and decreased muscle strength and other manifestations of femoral nerve injury were observed. Postoperative X-ray examination showed that the focus had no recurrence, the primary reactive hyperplastic bone was absorbed by itself, and the morphology of femur returned to normal (Figure 4).

## Discussion

OO is a benign bone tumour with pain as the first symptom accompanied by evident nocturnal pain and typical intra-cortical nidus surrounded by sclerosis and cortical thickening as the primary manifestation, which often requires surgical intervention (2, 3, 25). The proximal femoral constitutes the most susceptible part for OO, which is challenging to treat because of its deep location, close to the hip joint, and complex local anatomy (7, 26, 27). Presently, the main surgical treatment methods for OO include open surgical resection and minimally invasive treatment, such as CT guided radiofrequency ablation (RFA) (11, 12, 15), cryoablation (28), and microwave ablation (29). Whatever the kind of treatment adopted, the key to successful surgical treatment of OO is based on the accurate location of the nidus and its subsequent complete removal (7, 18, 19). Minimally invasive surgery has the advantages of less trauma, precise location, and short operation time. However, it requires high hardware conditions, high technical operation requirements, presents with incomplete removal of the nidus, easy to damage adjacent tissues, and unable to carry out a pathological examination, and among other limitations, which affect its popularization and application (2, 16, 30). At the same time, minimally invasive surgery is significantly inferior to open surgical resection in terms of OO recurrence rate and incidence of complications (9, 11, 28, 31, 32). The trauma associated with open surgery is relatively higher but wholly and accurately results in the removal of the nidus, improving the positive rate of pathological examination, and reduces the postoperative recurrence rate. Synovium cleaning and local soft tissue loosening are conducted where necessary, and bone grafting is performed for large bone defects, to reduce the risk of postoperative fractures. Given that the proximal femoral is close to the hip joint, the local anatomical structure is complex, and it is close to the important neurovascular femoral nerve, we speculated that open surgical resection is more suitable for the treatment of proximal femoral OO.

In the cohort, 35 patients were treated using open surgical resection and SP approach was selected aid in exposing the foci (Figure 3). This surgical approach completely avoids the anterior femoral arteriovenous and femoral nerves, and fully exposes the lesion of the proximal femoral without affecting blood supply to the femoral head, so as to clear the nidus under direct vision. During the follow-up period, none of the patients in this group had recurrence postoperatively, nor signs of femoral nerve injury such as decreased muscle strength. Additionally, deep vein thrombosis and femoral head necrosis were not observed. Postoperative VAS score and modified Harris score were significantly improved compared with preoperative.

The healing effect of open surgical resection on OO is highly effective, but the diagnosis of OO at proximal femoral is challenging; hence requires further investigations. In our study, the preoperative misdiagnosis rate was 42.9%, and a significant number of the patients underwent multiple surgical procedures due to misdiagnosis. This causes considerable physical suffering and financial burden. Through comparative analysis, we found that the complex and diverse clinical manifestations of proximal femoral OO cause its objective misdiagnosis, whereas lack of clear understanding of the disease and selection of the wrong method of examination constitute the frequent subjective causes of misdiagnosis (8, 22, 33). OO is characterized by persistent pain, accompanied by nocturnal pain, which is relieved by oral use of NSAIDs. However, OO in the proximal femoral is associated with joint cavity effusion, bone marrow edema, and soft tissue swelling. These nonspecific inflammatory reactions increase the pressure in the joint cavity, leading to changes in the property of the pain. The disappearance of postoperative pain and the improvement of hip joint function are the direct evidence for the effectiveness of surgical treatment. Most patients have limited hip joint activity due to preoperative pain and inflammatory reaction of hip joint. Once the focus is cleared, the human body will start the repair mechanism, the original pain will disappear, the periosteal reaction will gradually shape, and the inflammatory reaction of hip joint will gradually improve. Therefore, patients can get an intuitive curative effect. Furthermore, considering that the first visit of most patients to the doctor comprise of non-osteooncologists, even with typical clinical manifestations, proximal femoral OO is easily ignored. The tiny nidus in the early stage and the inconspicuous surrounding osteosclerosis make inexperienced radiologists overlook the possibility of OO, which additionally results in misdiagnosis or missed diagnosis. At the same time, MRI is widely used as the preferred method of examining joints as patients mostly present with hip joint pain. The tiny nidus lacks characteristic signals, and the spatial resolution of MRI is relatively low in addition to being sensitive to joint swelling, fluid accumulation, and bone marrow abnormalities, which easily attract the radiology reader's attention affecting the diagnosis. On the contrary, thin-layer CT has an optimal spatial resolution, accurately displaying the nidus and abnormal calcifications, especially for sites with complex anatomical structures. Therefore, thin-layer CT constitutes the most valuable method for the diagnosis of OO.

The clinical and imaging manifestations of proximal femoral OO are not necessarily representative. There could be significant differences in the performance of patients during different periods. Therefore, proximal femoral OO should be clearly distinguished from the following diseases at the diagnosis (4, 5, 9): ① Sclerosing osteomyelitis whose radiography manifestations include symmetric thickening and sclerosis of the bilateral bone cortex with no nidus transparent area. The pain is intermittent with no nocturnal pain, and salicylic acid is ineffective. ② Hip impingement syndrome, which is mainly manifested by groin pain, no nocturnal pain, more obvious during exercise or squat, and aggravated symptoms during hip flexion and internal rotation. Physical examination showed that the impact test was positive, MRI showed spotted subchondral injury in the anterior upper part of the femoral head, and acetabular glenoid lip injury. ③ Chronic localized bone abscess disease that is prone to the epiphysis of the long diaphysis, with evident inflammatory manifestations, including redness, swelling, heat, pain, and a history of repeated attacks, without the regular pain of OO. ④ Synovitis of the hip joint, which often occurs in young children, and the symptoms are transient. The course of disease rarely exceeds three weeks. There is a history of violent activity before the onset, and the pain symptoms are quickly relieved after motionless rest. ⑤ Synovial tuberculosis of the hip typified by systemic tuberculosis poisoning with the radiography showing widening of the hip joint space. ⑥ Perthes disease characterized by hip pain and lameness as the primary symptoms, the femoral head presents with a crescent sign, and the necrosis of the femoral head may collapse.

Although the results of this study are satisfactory, there are still the following limitations. The small sample size poses a challenge of establishing the potential links between demographic, imaging or clinical features, and treatment failure or complications. Secondly, the use of NSAIDs may affect the preoperative VAS score, resulting in bias in the statistical analysis results. Third, the specific efficacy of this operation in the treatment of proximal femoral OO has no case-control and effective comparative analysis, further studies should be conducted using a large sample with a multicentre case-control study.

In summary, open surgical resection constitutes an effective method for the treatment of proximal femoral OO. Accurate and complete removal of the nidus is the core concept of this surgical treatment. Lack of clear understanding of the disease, wrong selection of examination methods, and the complexity and diversity of its clinical manifestations constitute the primary reasons for the misdiagnosis of proximal femoral OO.

## Data Availability

The original contributions presented in the study are included in the article/Supplementary Material, further inquiries can be directed to the corresponding author/s.
