# Supplementary material for: Osteoid Osteoma of the Proximal Femur: Pitfalls in Diagnosis and Performance of Open Surgical Resection
Source: Front Surg. 2022 Jun 28;9:922317. doi: 10.3389/fsurg.2022.922317 (PMC9273932; doi:10.3389/fsurg.2022.922317)
Supplement: Supplementary file 1 [file Table_2_v1.doc]

**Supplemental Tables: Demographic data of patients with OO**

| Patient | Gender | Age | Follow-up time(months) | Classification | Localization | Duration of symptom (months) | Misdiagnosis* | Preop-VAS score | Postop-VAS score | Preop-Harris score | Postop-Harris score |
| --- | --- | --- | --- | --- | --- | --- | --- | --- | --- | --- | --- |
| 1 | F | 20 | 73 | intracortical | extra-articular | 3 | / | 5 | 0 | 44 | 100 |
| 2 | F | 18 | 81 | intracortical | extra-articular | 2 | / | 7 | 1 | 53 | 100 |
| 3 | M | 25 | 60 | intracortical | extra-articular | 13 | HIS | 7 | 2 | 37 | 100 |
| 4 | M | 13 | 54 | intracortical | intra-articular | 5 | / | 4 | 0 | 54 | 100 |
| 5 | M | 13 | 48 | intracortical | extra-articular | 9 | SO | 6 | 0 | 58 | 98 |
| 6 | F | 14 | 59 | intracortical | extra-articular | 3 | / | 6 | 0 | 67 | 100 |
| 7 | M | 15 | 58 | intracortical | extra-articular | 3 | / | 7 | 0 | 60 | 100 |
| 8 | M | 21 | 58 | intracortical | intra-articular | 5 | / | 7 | 0 | 48 | 97 |
| 9 | M | 13 | 57 | subperiosteal | extra-articular | 1 | / | 6 | 0 | 55 | 100 |
| 10 | F | 22 | 51 | medullary | intra-articular | 2 | / | 5 | 0 | 34 | 99 |
| 11 | M | 14 | 48 | intracortical | extra-articular | 11 | HIS | 6 | 1 | 77 | 100 |
| 12 | M | 15 | 46 | medullary | intra-articular | 2 | / | 6 | 1 | 61 | 100 |
| 13 | M | 14 | 33 | subperiosteal | extra-articular | 8 | synovitis | 6 | 0 | 53 | 98 |
| 14 | M | 14 | 45 | intracortical | extra-articular | 3 | / | 5 | 0 | 54 | 100 |
| 15 | M | 15 | 34 | intracortical | extra-articular | 4 | / | 4 | 0 | 58 | 100 |
| 16 | M | 13 | 28 | intracortical | extra-articular | 7 | synovitis | 5 | 0 | 65 | 100 |
| 17 | F | 15 | 30 | medullary | intra-articular | 2 | / | 6 | 1 | 47 | 100 |
| 18 | M | 17 | 31 | intracortical | extra-articular | 14 | synovitis | 5 | 0 | 56 | 97 |
| 19 | M | 14 | 28 | intracortical | extra-articular | 10 | INI | 7 | 2 | 57 | 100 |
| 20 | F | 17 | 27 | intracortical | extra-articular | 9 | JT | 6 | 0 | 58 | 98 |
| 21 | M | 14 | 47 | medullary | intra-articular | 5 | / | 7 | 1 | 39 | 100 |
| 22 | M | 24 | 41 | intracortical | intra-articular | 8 | INI | 6 | 0 | 43 | 100 |
| 23 | F | 16 | 27 | subperiosteal | extra-articular | 9 | SO | 4 | 0 | 46 | 100 |
| 24 | M | 22 | 26 | intracortical | intra-articular | 3 | / | 5 | 0 | 48 | 100 |
| 25 | M | 15 | 25 | intracortical | intra-articular | 2 | / | 5 | 0 | 55 | 99 |
| 26 | M | 13 | 33 | subperiosteal | extra-articular | 9 | PD | 7 | 1 | 68 | 100 |
| 27 | F | 14 | 29 | intracortical | intra-articular | 3 | / | 6 | 1 | 66 | 98 |
| 28 | M | 15 | 32 | intracortical | extra-articular | 11 | HIS | 5 | 0 | 61 | 100 |
| 29 | F | 15 | 24 | intracortical | extra-articular | 10 | SO | 7 | 0 | 35 | 100 |
| 30 | M | 13 | 43 | intracortical | extra-articular | 6 | / | 5 | 2 | 55 | 100 |
| 31 | F | 16 | 31 | intracortical | extra-articular | 8 | / | 4 | 1 | 67 | 99 |
| 32 | M | 17 | 28 | intracortical | extra-articular | 4 | HIS | 6 | 0 | 78 | 100 |
| 33 | M | 22 | 27 | intracortical | extra-articular | 9 | / | 5 | 0 | 67 | 98 |
| 34 | M | 13 | 37 | medullary | intra-articular | 12 | HIS | 4 | 2 | 56 | 100 |
| 35 | F | 15 | 51 | medullary | extra-articular | 6 | / | 4 | 2 | 67 | 100 |

*SO, sclerosing osteomyelitis; INI, intra-articular infection; PD, perthes disease; JT, joint tuberculosis; HIS, hip impingement syndrome
